# Antiphospholipid antibodies as potential predictors of disease severity and poor prognosis in systemic lupus erythematosus-associated thrombocytopenia: results from a real-world CSTAR cohort study

**DOI:** 10.1186/s13075-024-03305-w

**Published:** 2024-03-12

**Authors:** Jun Li, Liying Peng, Lijun Wu, Yufang Ding, Xinwang Duan, Jian Xu, Wei Wei, Zhen Chen, Cheng Zhao, Min Yang, Nan Jiang, Shangzhu Zhang, Qian Wang, Xinping Tian, Mengtao Li, Xiaofeng Zeng, Yan Zhao, Jiuliang Zhao

**Affiliations:** 1 Department of Rheumatology and Clinical Immunology, Peking Union Medical College Hospital, Peking Union Medical College, National Clinical Research Center for Dermatologic and Immunologic Diseases (NCRC-DID), Ministry of Science & Technology, State Key Laboratory of Complex Severe and Rare Diseases, Key Laboratory of Rheumatology and Clinical Immunology, Chinese Academy of Medical Sciences, Peking Union Medical College Hospital, Ministry of Education, Beijing, 100730 China; 2 Department of Rheumatology and Immunology, People’s Hospital of Xinjiang Uygur Autonomous Region, Urumqi, 830001 China; 3 Department of Rheumatology, The Second Affiliated Hospital of Nanchang University, Nanchang, 330006 China; 4 Department of Rheumatology and Immunology, First Affiliated Hospital of Kunming Medical University, Kunming, 650032 China; 5 Department of Rheumatology and Immunology, Tianjin Medical University General Hospital, Tianjin, 300052 China; 6 Department of Rheumatology, The Second Affiliated Hospital of Fujian Medical University, Quanzhou, 362000 China; 7 Department of Rheumatology and Immunology, The People’s Hospital of Guangxi Zhuang Autonomous Region, Nanning, 530021 China; 8 Department of Rheumatic & TCM Medical Center, Nanfang Hospital, Southern Medical University, Guangzhou, 510515 China

**Keywords:** Thrombocytopenia, Systemic Lupus Erythematosus, Antiphospholipid antibodies, Severity, Prognosis

## Abstract

**Background:**

To investigate the role of antiphospholipid antibodies (aPLs) in the disease severity and prognosis of SLE-related thrombocytopenia (SLE-TP).

**Methods:**

This multicenter prospective study was conducted based on data from the CSTAR registry. TP was defined as a platelet count<100 × 10^9^/L. Demographic characteristics, platelet count, clinical manifestations, disease activity, and autoantibody profiles were collected at baseline. Relapse was defined as the loss of remission. Bone marrow aspirate reports were also collected.

**Results:**

A total of 350 SLE-TP patients with complete follow-up data, 194 (55.4%) were aPLs positive. At baseline, SLE-TP patients with aPLs had lower baseline platelet counts (61.0 × 10^9^/L vs. 76.5 × 10^9^/L, *P*<0.001), and a higher proportion of moderate to severe cases (24.2% vs. 14.1% ; 18.0% vs. 8.3%, *P*<0.001). SLE-TP patients with aPLs also had lower platelet counts at their lowest point (37.0 × 10^9^/L vs. 51.0 × 10^9^/L, *P* = 0.002). In addition, thean increasing number of aPLs types was associated with a decrease in the baseline and minimum values of platelets ( *P*<0.001, *P* = 0.001). During follow-up, SLE-TP carrying aPLs had a higher relapse rate (58.2% vs. 44.2%, *P* = 0.009) and a lower complete response (CR) rate. As the types of aPLs increased, the relapse rate increased, and the CR rate decreased. Furthermore, there was no significant difference in the ratio of granulocytes to red blood cells (G/E), the total number of megakaryocyte and categories.

**Conclusion:**

SLE-TP patients with positive aPLs had more severe disease a lower remission rate but a higher relapse rate.

## Background

Systemic lupus erythematosus (SLE) is a chronic complex systemic autoimmune disease involving multiple organs with multiple autoantibodies. Thrombocytopenia (TP) is a common clinical haematological abnormality, which is also known as one of the hematological criteria of SLE, according to the American College of Rheumatology (ACR) classification criteria [1]. Severe thrombocytopenia is correlated with disease activity and a worse prognosis of SLE-associated thrombocytopenia (SLE-TP) [2, 3]. Antiphospholipid antibodies (aPLs) have been proven to cause thrombocytopenia through various mechanisms [4]. However, the mechanisms of thrombocytopenia in SLE patients are not fully known and may involve the production, destruction, and distribution of platelets. Existing studies have confirmed that aPL antibodies can cause thrombocytopenia through multiple mechanisms [5], but the impact of aPL antibodies on SLE-TP remains a matter of debate. Therefore, the aim of this multicenter prospective study was to investigate the role of aPL antibodies in the disease severity and prognosis of SLE-TP.

## Methods

### Patients and follow-up

The Chinese SLE Treatment and Research Group (CSTAR) registry is a nationwide online registry database that was funded by the Chinese Ministry of Science & Technology in 2009 [6] and has comprehensively described the major demographic, clinical manifestations, and laboratory measurements of SLE patients [7]. Based on this prospective cohort, we consecutively collected SLE patients with thrombocytopenia from January 2012 to April 2023. All SLE patients fulfilled either the 2012 classification criteria of the Systemic Lupus International Collaborating Centers (SLICC) group [8] or the 2019 American College of Rheumatology (ACR)/European League Against Rheumatism (EUALR) classification criteria for SLE [1]. Thrombocytopenia was defined as a platelet count of < 100 × 10^9^/L [9]. Patients with other causes of thrombocytopenia, lost to follow-up or incomplete data were excluded. The baseline time is defined as the time when thrombocytopenia first occurs after the diagnosis of SLE. We collected baseline data through the CSTAR online registry, including demographics, clinical manifestations, laboratory data and SLE Disease Activity Index 2000 (SLEDAI-2k). Continuous follow-up included changes in platelet count, treatment medication status, clinical manifestations and laboratory indices. Bone marrow aspirate was obtained to confirm impairment of megakaryocyte maturation. Patients carrying at least one type of aPLs were classified as the aPLs positive group. This study was approved by the medical ethics committee of Peking Union Medical College Hospital (Approval number, JS-3386D).

### Definitions

SLE-TP patients were classified into three groups based on the degree of low platelet count: mild (platelet count between 50 and 100 × 10^9^/L), moderate (platelet count between 20 and 50 × 10^9^/L), and severe (platelet count less than 20 × 10^9^/L). Treatment response of thrombocytopenia was defined according to the guidelines of immune thrombocytopenia of the American Society of Hematology, endorsed by the Scientific Working Group on Thrombocytopenias of the European Hematology Association (EHA) [10]. Specifically, “Complete response” (CR) was defined as platelet count recovered to at least 100 × 10^9^/L. “Response” (R) was defined as the restoration of the platelet count to a range between 30 and 100 × 10^9^/L, along with at least a twofold increase from the baseline count. “No response” (NR) was recorded when the platelet count remained below 30 × 10^9^/L or did not double from the baseline count. “Loss of CR or R” was denoted by a platelet count falling below 100 × 10^9^/L (for those in CR), or dropping below 30 × 10^9^/L or failing to achieve at least a twofold increase from the baseline count (for those in R).

### Measures

Anti-nuclear antibody (ANA) was detected by indirect immunofluorescence (IIF) assay with the Hep-2 cell line from Euroimmun AG (Lübeck, Germany). Anti-dsDNA antibody was tested by IIF using flagellate protoctista substrates and enzyme-linked immunosorbent assay (ELISA) using IMTEC ds-DNA Antibodies ELISA KT (Human Worldwide, Wiesbaden, Germany). Anti-extractable nuclear antigen (ENA) antibodies were tested with the immunoblotting assay using the EUROLINE ENA Profile 9 Ag (Euroimmun) according to the manufacturer’s instructions. Anti-RibP antibodies were identified by immunoblotting containing all three native RibP (P0, P1, P2) antigenic proteins. The aPLs, including IgG or IgM anticardiolipin antibodies, anti-𝛽2 glycoprotein I, and lupus anticoagulant. Levels of anticardiolipin (aCL) and anti-b2glycoprotein (GP) I antibody isotypes were quantified by QUANTA LiteTM ELISA kits provided by INOVA diagnostics, Inc. (San Diego, CA, USA). The defined cutoff values were 40 GPL/MPL. LA was measured by dilute Russel viper venom time (dRVVT), with a ratio above 1.20 considered positive. The manufacturer’s recommendations were followed carefully.

### Statistical analyses

The Shapiro–Wilk test was performed to assess normality. Continuous variables were expressed as medians and 25th and 75th percentiles [quantile 1 (Q1) and Q3, respectively], while categorical variables were presented as numbers and percentages. For comparisons of categorical variables, we used Chi-square (χ^2) and Fisher’s exact tests, and for continuous variables, independent t tests were employed. Data not following a normal distribution were compared using the Mann‒Whitney U test. A *p* value < 0.05 (two-sided) was considered statistically significant. Statistical analyses were performed using SPSS 24.0 (IBM, Armonk, NY, USA).

## Results

### Baseline characteristics of SLE-TP patients

**Fig. 1:**
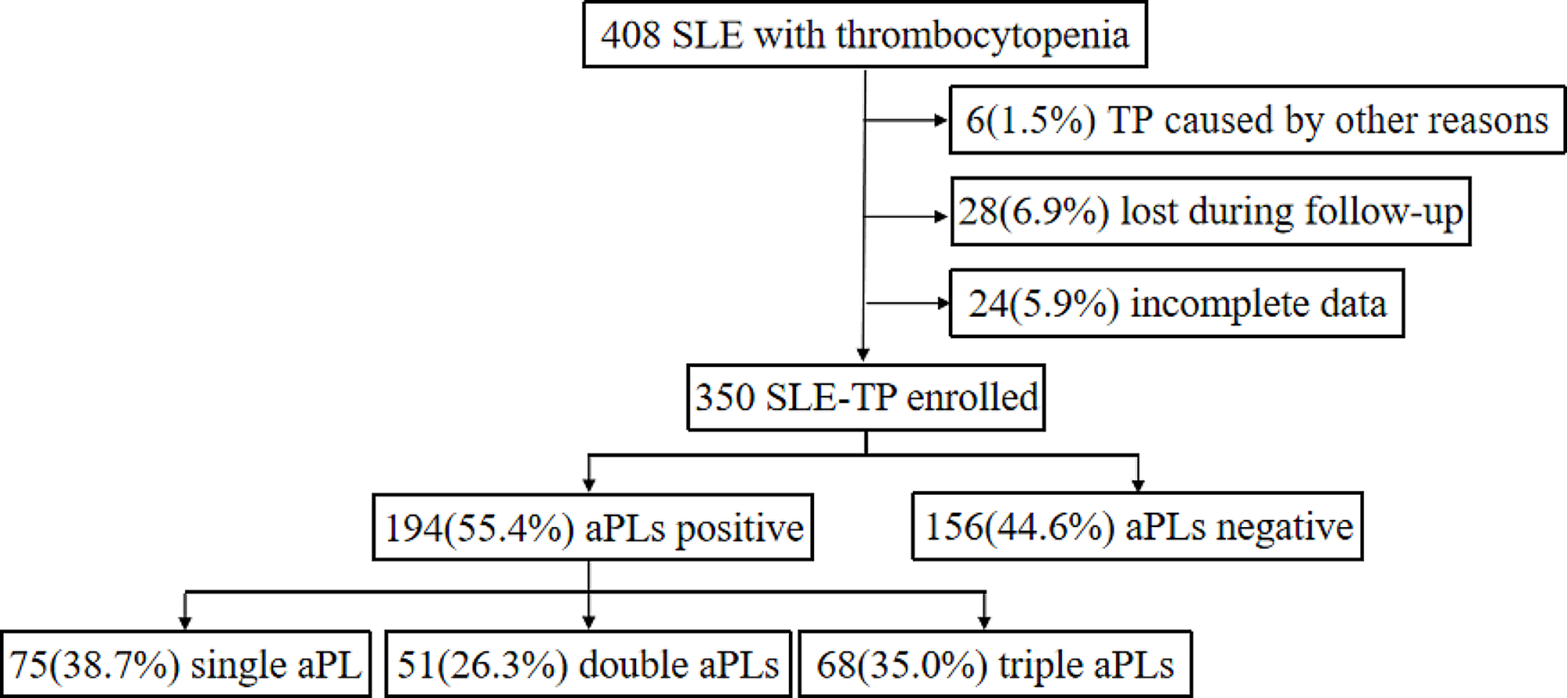
Flow chart of patient selection. A total of 408 SLE patients with thrombocytopenia (SLE-TP) in the Chinese SLE Treatment and Research Group(CSTAR). Six thrombocytopenia patients were caused by other reasons. 28 were lost during follow-up and 24 had incomplete data. 350 SLE-TP with complete platelet follow-up data were included in this cohort, of whom 156 without any positive aPLs. Among 194 SLE-TP with aPLs, 75 had single kind aPL, 51 had double aPLs, and 68 had triple aPLs

This cohort consecutively enrolled 350 SLE-TP patients. Six (1.5%) patients with other causes of thrombocytopenia were excluded: three due to splenectomy for hypersplenism, two with chronic myeloid leukemia and one with acute myeloblastic leukemia. 28 (6.9%) patients who were lost during follow-up and 24 (5.9%) patients with incomplete data were also excluded. Among the 350 SLE-TP patients, 194 (55.4%) carried at least one type of aPLs and were classified as the aPLs positive group: 75 (38.7%) had single aPL, 51 (26.3%) had double aPLs, and 68 (35.0%) had triple aPLs. The aPLs negative group consisted of 156 (44.6%) without any positive aPLs (Fig. 1). The overall median follow-up time was 5.7 [3.8, 8.2] years. The baseline demographics, profile of autoantibodies and clinical characteristics are presented in Table 1. Gender distribution, age, and disease duration were similar between aPLs positive and aPLs negative patients. Among them, a total of 49 SLE patients with APS (14.0%) were all aPLs positive carriers. In addition, a total of 44 patients (12.6%) experienced thrombotic events, of which 37/194 (19.1%) were positive for APLs and 7/156 (4.5%) were negative for aPL (*P* < 0.001).

**Table 1:** Baseline characteristics, profile of autoantibodies, and clinical manifestations of 350 SLE-TP patients

	Total(*n* = 350)	aPLs+(*n* = 194)	aPLs-(*n* = 156)	*P*
Female gender, n(%)	313(89.4)	194 (86.6)	145 (92.9)	0.055
Age (years)	33 (27,43)	33 (27,44)	33(26,42)	0.939
Disease duration (years)	10.0(6.7,14.3)	9.6 (5.6,13.9)	10.8 (7.2,14.7)	0.321
SLEDAI-2k	3(1,7)	3(1,8)	2(1,7)	0.282
Baseline platelet count (×10^9^/L)	68.5(40,90)	61(33,86)	76.5(54, 90)	**<0.001**
Severe (<20 × 10^9^/L), n(%)	48(13.7)	35(18.0)	13(8.3)	
Moderate (20–50 × 10^9^/L), n(%)	69(19.7)	47(24.2)	22(14.1)	**<0.001**
Mild (50–100 × 10^9^/L), n(%)	233(66.6)	112(57.7)	121(77.6)	
Skin and mucous membranes, n(%)	149(42.6)	77(39.7)	72(46.2)	0.224
Arthritis, n(%)	124(35.4)	58(29.9)	66(42.3)	**0.016**
Serositis, n(%)	68(19.4)	29(14.9)	39(25.0)	**0.018**
Neurological involvement, n(%)	63(18.0)	38(19.6)	25(16.0)	0.389
Nephropathy, n(%)	140(40.0)	72(37.1)	68(43.6)	0.219
HGB (×10^12^/L)	119.5(102,135)	119(102,137)	120.5(104,133)	0.795
Anti-double stranded DNA (anti-dsDNA) antibody, n(%)	92(26.3)	42(21.8)	50(32.1)	**0.028**
Anti-Sm antibody, n(%)	85(24.3)	39(20.1)	46(29.5)	**0.042**
Anti-RNP antibody, n(%)	145(41.4)	64(33.0)	81(51.9)	**<0.001**
Anti-SSA antibody, n(%)	204(58.3)	105(54.1)	99(63.5)	0.078
Anti-SSB antibody, n(%)	50(14.3)	23(11.9)	27(17.3)	0.147
Anti-ribosomal P protein (anti-RibP) antibody, n(%)	78(22.3)	45(23.2)	33(21.2)	0.648
Anti-nucleosome antibody (ANuA), n(%)	48(13.7)	33(17.0)	15(9.6)	**0.046**
Anti-histone antibody (AHA), n(%)	58(16.6)	37(19.1)	21(13.5)	0.161
APS, n(%)	49(14.0)	49(29.3)	0	**<0.001**
Thrombosis, n(%)	44(12.6)	37(19.1)	7(4.5)	**<0.001**
Glucocorticoids, n(%)	350 (100)	194 (100)	156 (100)	1
Immunosuppressant, n(%)	305 (87.1)	169 (87.1)	136 (87.2)	0.985
Anticoagulant therapy, n(%)	59 (16.9)	46 (23.7)	13 (8.3)	**<0.001**
Antiplatelet therapy, n(%)	128 (36.6)	79 (40.7)	49 (31.4)	0.072

### The association between aPLs and severity of SLE-TP

Compared with SLE-TP patients without aPLs, those with positive aPLs had lower baseline platelet counts (61.0 × 10^9^/L vs. 76.5 × 10^9^/L, *P* < 0.001) and a higher proportion of moderate and severe cases (42.2% vs. 22.4%, *P* < 0.001). In addition, SLE-TP patients with aPLs had lower platelet counts at their lowest point (37.0 × 10^9^/L vs. 51.0 × 10^9^/L, *P* = 0.002) during the follow-up.

### **The association between aPLs and treatment response of thrombocytopenia**

**Fig. 2:**
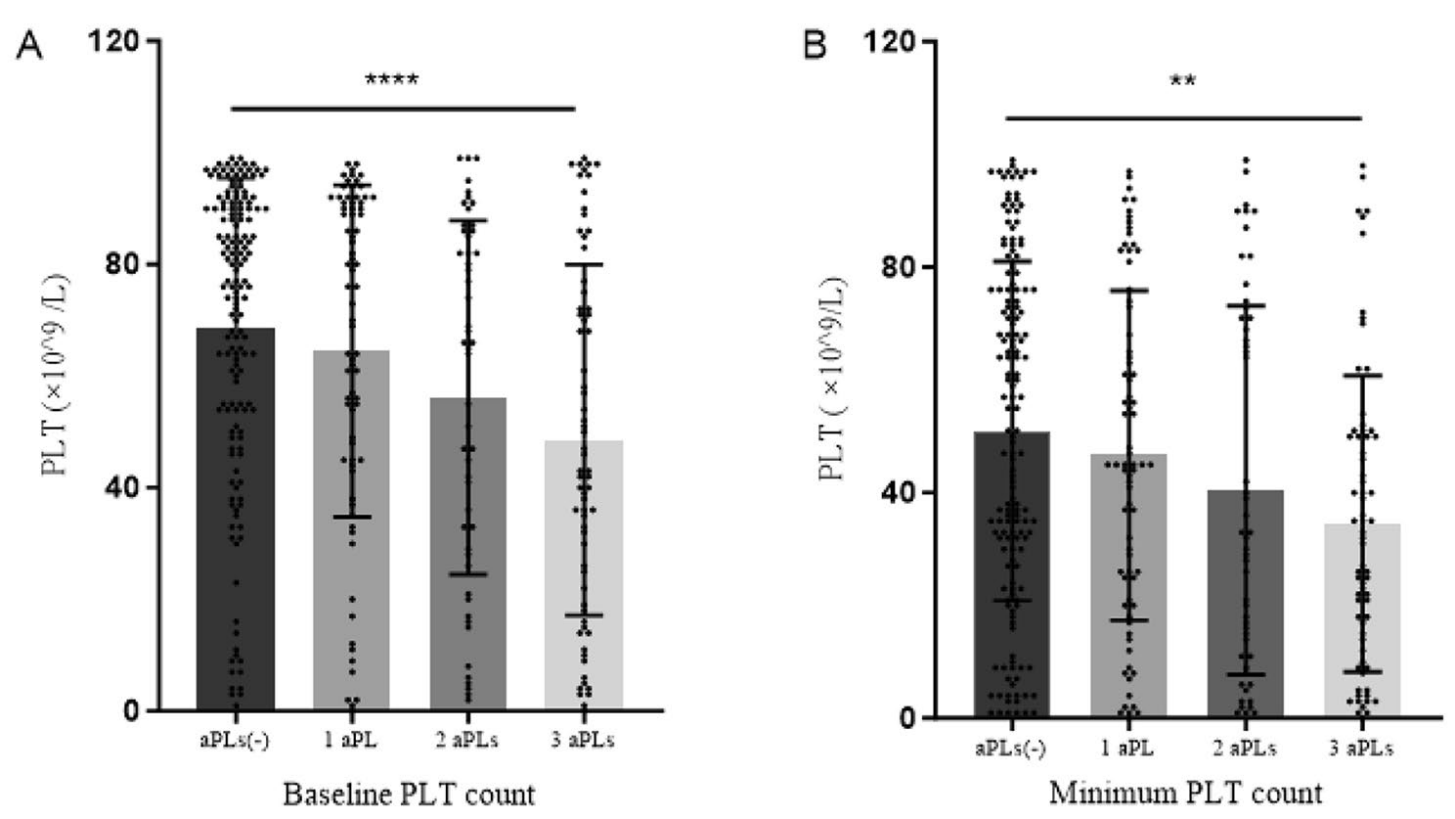
**(A)**. Baseline platelet count of SLE-TP with different aPLs. **(B)** Minimum platelet count of SLE-TP with different aPLs

The increasing number of aPLs types was associated with a decrease in the baseline and minimum values of platelets (*P* < 0.001; *P =* 0.001) (Fig. 2). Meanwhile, SLE-TP patients carrying aPLs had a lower complete response (CR) rate, and as the number of positive types of aPLs increased, the CR rate showed a downward trend (92.0%, 82.4%, and 80.9%, respectively). Notably, SLE-TP patients with aPLs had a significantly higher loss of CR/R rate (58.2% vs. 44.2%, *P* = 0.009) and showed an upward trend as the number of positive types of aPLs increased (54.7%, 54.9%, 64.7%, respectively) (Table 2).

**Table 2:** The minimum PLT value during follow-up and the treatment response of 350 SLE-TP patients with different aPLs

	aPLs+(*n* = 194)				aPLs- (*n* = 156)	*P*
	1 aPL+ (*n* = 75, 38.7%)	2 aPLs+ (*n* = 51, 26.2%)	3 aPLs+ (*n* = 68, 35.1%)	aPLs + total		
Minimum platelet count (×10^9^/L)	45(21,73)	32(11,71)	31.5(12.5,50)	37(15,64)	51(27,76)	**0.002**
Severe(<20 × 10^9^/L), n(%)	16(21.3)	19(37.3)	22(32.4)	57(29.4)	28(17.9)	**0.010**
Moderate(20–50 × 10^9^/L), n(%)	27(36.0)	13(25.5)	26(38.2)	66(34.0)	48(30.8)	
Mild(50–100 × 10^9^/L), n(%)	32(42.7)	19(37.3)	20(29.4)	71(36.6)	80(51.3)	
Latest platelet count (×10^9^/L)	125(85,211)	147(90,222)	100.5(64,172)	123(79,209)	141(82,212)	0.361
CR, n(%)	69(92.0)	42(82.4)	55(80.9)	166(85.6)	135(86.5)	
R, n(%)	1(1.3)	2(3.9)	7(10.3)	10(5.2)	1(0.6)	**0.021**
NR, n(%)	5(6.7)	7(13.7)	6(8.8)	18(9.3)	20(12.8)	
Loss of CR or R, n(%)	41(54.7)	28(54.9)	44(64.7)	113(58.2)	69(44.2)	**0.009**

Note:CR, complete response,is defined as platelet count recovered to at least 100 × 10^9^/L. R, response, is defined as the restoration of the platelet count to a range 30 and 100 × 10^9^/L and along with at least a twofold increase from the baseline count. NR, nonresponse,is recorded when the platelet count remained below 30 × 10^9^/L or did not double from the baseline count. Loss of CR or R, is denoted by a platelet count falling below 100 × 10^9^/L (for those in CR), or dropping below 30 × 10^9^/L or failing to achieve at least a twofold increase from the baseline count (for those in R)

### The association between aPLs and bone marrow aspirate

The correlation analysis of 61 SLE-TP (41 aPLs positive, 67.2%) cases with qualified bone marrow aspirate reports showed that there was no significant difference in the ratio of granulocytes to red blood cells and the total number of megakaryocytes (*P* = 0.736, *P* = 0.380, respectively). Furthermore, the various classifications of megakaryocytes, including granular cells, nude cells and production plate cells, also had similar results (*P* = 0.360, *P* = 0.250, *P* = 0.381, respectively) (Table 3).

**Table 3:** Bone marrow aspirate report of 61 SLE-TP patients

	aPLs+(*n* = 41)	aPLs-(*n* = 20)	*P*
Ratio of granulocytes to red blood cells, mean ± S.D.	3.1 ± 2.2	2.9 ± 2.6	0.736
Total megakaryocyte count, mean ± S.D.	64.3 ± 79.3	46.2 ± 57.8	0.380
Granular cell ratio, mean ± S.D.	90.1 ± 17.1	86.0 ± 13.6	0.360
Nude cell ratio, mean ± S.D.	6.1 ± 8.0	9.6 ± 11.5	0.250
Production plate cell ratio, mean ± S.D.	0.7 ± 1.5	1.3 ± 2.8	0.381

## Discussion

In this multicenter prospective cohort study, we explored the impact of aPLs on SLE-TP by comparing SLE-TP patients with positive and negative aPLs. We found that SLE-TP patients with aPLs exhibited lower platelet levels, both at baseline and minimum counts, and had a higher relapse rate. Given the increasing recognition of thrombocytopenia’s role in mortality and end-organ damage risk in SLE patients, monitoring and predicting thrombocytopenia efficacy have received increasing attention. The associations discovered in our study suggest that aPLs may serve not only as predictors of thrombocytopenia severity but also as indicators of relapse rates. This suggests that in clinical practice, SLE-TP patients with positive aPLs encounter greater challenges in treatment and should receive more frequent follow-up to prevent recurrence.

Thrombocytopenia is a common manifestation of blood system involvement in SLE patients, with an incidence rate of 7–30% [11]. The pathophysiological mechanisms underlying thrombocytopenia in SLE patients are not fully known, but at least three mechanisms have been identified: impaired production of platelets in the bone marrow, sequestration of platelets in the spleen, or accelerated destruction of platelets in the peripheral circulation [12]. aPLs may play an important role in these processes [13, 14].While it is not officially classified as a criterion, a reduced platelet count is a frequently observed laboratory characteristic in patients with anti-phospholipid syndrome (APS), regardless of whether they have a concurrent diagnosis of SLE. aPLs, which can appear in SLE and APS patients, are a heterogeneous group of autoantibodies reacting against phospholipids, phospholipid-protein complexes, and phospholipid-binding proteins, including lupus anticoagulant (LA), anticardiolipin (aCL) and anti-beta2 glycoprotein I (anti-β2GP1) antibodies [15]. Studies have shown that aPLs may cause thrombocytopenia through various mechanisms: aPLs bind phospholipids on platelet membranes or endothelial cells in a cross reaction, and antibody-opsonized platelets are recognized, phagocytized, and destroyed through Fcγ receptors by macrophages in the spleen, liver and bone marrow [16]. Additionally, aPLs can activate complement through classical pathways, directly mediating platelet destruction [17, 18]. Furthermore, in addition to activating the traditional p38/MAPK (mitogen activated protein kinase, MAPK) signaling pathway [19], recent research has shown that aPLs may also overactivate the mTORC2 (mammalian target of the rapamycin complex 2)/Akt pathway, inducing platelet activation and decreasing platelet count [20]. Our study corroborates the clinical medical perspective on the association between aPLs and SLE-TP.

Despite the increasing attention to the harm caused by severe and recurrent low platelet counts in SLE patients, there is currently no internationally recognized management and effective predictive indicator for SLE-TP. In our study, SLE-TP patients with positive aPLs had lower platelet counts during follow-up, and the relapse rate was higher. Moreover, the severity and relapse rate of thrombocytopenia were positively correlated with the number of types of aPLs, suggesting that aPLs may serve as valuable indicators for disease severity, treatment response and prognosis in SLE-TP patients.

Our study has several limitations. Firstly, due to a relatively small sample size, and the fact that only a subset of patients underwent testing for the types of aPLs, we were unable to investigate the impact of different types of aPLs on platelet reduction. Secondly, a limited number of patients underwent bone marrow aspiration, which hindered our exploration of the effects of aPLs on the platelet production process, such as whether aPLs affect the quantity and maturation of megakaryocytes. Thirdly, comprehensive records of bleeding events in SLE-TP patients were not available, thus preventing a thorough investigation into the influence of aPLs on bleeding occurrences.

## Conclusions

In conclusion, our study demonstrated that SLE-TP patients with positive aPLs exhibited a more severe disease condition and a higher relapse rate. These findings indicate that aPLs may serve as valuable indicators for disease severity and prognosis in SLE patients with thrombocytopenia. Therefore, implementing proactive management strategies and maintaining vigilance toward relapse are essential for SLE-TP patients with positive aPLs.

## Data Availability

No datasets were generated or analysed during the current study.
